# Ectopic Expression of Vaccinia Virus E3 and K3 Cannot Rescue Ectromelia Virus Replication in Rabbit RK13 Cells

**DOI:** 10.1371/journal.pone.0119189

**Published:** 2015-03-03

**Authors:** Erin S. Hand, Sherry L. Haller, Chen Peng, Stefan Rothenburg, Adam R. Hersperger

**Affiliations:** 1 Department of Biology, Albright College, Reading, Pennsylvania, United States of America; 2 Laboratory for Host-Specific Virology, Division of Biology, Kansas State University, Manhattan, Kansas, United States of America; University of Texas HSC at San Antonio, UNITED STATES

## Abstract

As a group, poxviruses have been shown to infect a wide variety of animal species. However, there is individual variability in the range of species able to be productively infected. In this study, we observed that ectromelia virus (ECTV) does not replicate efficiently in cultured rabbit RK13 cells. Conversely, vaccinia virus (VACV) replicates well in these cells. Upon infection of RK13 cells, the replication cycle of ECTV is abortive in nature, resulting in a greatly reduced ability to spread among cells in culture. We observed ample levels of early gene expression but reduced detection of virus factories and severely blunted production of enveloped virus at the cell surface. This work focused on two important host range genes, named E3L and K3L, in VACV. Both VACV and ECTV express a functional protein product from the E3L gene, but only VACV contains an intact K3L gene. To better understand the discrepancy in replication capacity of these viruses, we examined the ability of ECTV to replicate in wild-type RK13 cells compared to cells that constitutively express E3 and K3 from VACV. The role these proteins play in the ability of VACV to replicate in RK13 cells was also analyzed to determine their individual contribution to viral replication and PKR activation. Since E3L and K3L are two relevant host range genes, we hypothesized that expression of one or both of them may have a positive impact on the ability of ECTV to replicate in RK13 cells. Using various methods to assess virus growth, we did not detect any significant differences with respect to the replication of ECTV between wild-type RK13 compared to versions of this cell line that stably expressed VACV E3 alone or in combination with K3. Therefore, there remain unanswered questions related to the factors that limit the host range of ECTV.

## Introduction

Ectromelia virus (ECTV; also referred to as “mousepox virus”) is a double-stranded DNA virus in the *Poxviridae* family. ECTV typically infects mice through abrasions in the skin [1]. Following initial replication at the site of infection, the virus disseminates to multiple organs over the course of several days [2]. Among the mice that survive the initial infection, characteristic pock lesions manifest on the skin [2,3] in a similar fashion to the disease manifestations of humans infected with variola virus (VARV), the causative agent of smallpox [4].

Vaccinia virus (VACV) is the best studied of all identified poxviruses and was used successfully in the global effort to eliminate circulating VARV. As with VARV and VACV, cross-immunity exists between ECTV and VACV [5,6]. Yet, despite a high degree of sequence identity between these two viruses [7], the courses of infection are quite distinct. While mice may be incidental hosts of VACV or even serve as reservoirs of the virus in the wild [8,9], it is fatal only under certain experimental conditions and routes of infection. In contrast, ECTV infection of susceptible mice (e.g. BALB/c strain) typically results in death even with a very low initial inoculum. Moreover, replication of VACV is restricted to the site of infection after cutaneous inoculation of mice [10], which is quite dissimilar from the infection course of ECTV.

As a group, poxviruses have been shown to infect a wide variety of animal species. However, at the level of individual members of this family, there is a profound variability in the host species range. Recently, there have been advances in our knowledge of poxvirus host range mechanisms [11–13] but overall the underlying molecular basis of these phenomena remains only partially understood. There have been about 12 different host range genes or gene families identified that contribute to poxvirus host range [14]. Interestingly, cowpox virus has the broadest host range of all known poxviruses and also contains the largest number (∼26–27 genes) of these host range genes [12]. In terms of the viruses examined in this study, VACV and ECTV possess 13 and 15 different intact host range genes, respectively [12]. The focus of this work is on the host range genes E3L and K3L (gene names based upon the nomenclature of the VACV-Copenhagen strain).

Both ECTV and VACV possess a gene of the E3L family, which encodes for a protein with an amino-terminal Z-DNA-binding domain and a carboxy-terminal double-stranded RNA (dsRNA)-binding domain [12,15]. The E3 protein of VACV has been shown to inhibit activation of protein kinase R (PKR), which is most likely a result of its ability to bind to dsRNA and prevent PKR homodimerization [16]. E3L can be an important host range gene since its deletion renders some poxviruses unable to replicate in cells derived from certain animal species (e.g. VACV with a deletion of E3L can no longer replicate in human HeLa or African green monkey Vero cells [17,18]). The E3L gene between VACV (Western Reserve) and ECTV (Moscow) shares 93% sequence identity [poxvirus.org]. The majority of the amino acid changes are found within the amino-terminal Z-DNA-binding domain but there are also some differences located in the dsRNA-binding domain [19]. It is possible that these variations in the amino acid sequence could affect the functionality of E3 within infected cells [20–23].

The protein encoded by the K3L gene is a molecular mimic of the eukaryotic translation initiation factor 2 alpha subunit (eIF2α) and acts to dampen the downstream effects of activated PKR [24]. Many poxviruses encode for a protein that falls within the K3L family, which suggests that this method of immune evasion is important during infection. Interestingly, ECTV does not encode a predicted functional version of K3 because its open reading frame contains a premature stop codon with deletions comprising residues previously reported to be essential for PKR inhibition [12,19,25].

As reported previously, we observed that ECTV does not replicate efficiently in cultured rabbit cells, such as RK13 [26]. Conversely, VACV replicates well in rabbit-derived cell lines. In this work, we sought to understand the reasons behind this discrepancy in more detail. We examined the ability of ECTV to replicate in wild-type RK13 cells compared to cells that had been engineered to stably express the VACV versions of E3L and K3L. Since these are two significant host range genes, we hypothesized that one or both of them may have a positive impact on ECTV’s ability to replicate in RK13 cells.

## Materials and Methods

### Cells and culture methods

European rabbit cell line RK13 (ATTC# CCL-37) and its derivatives RK13+E3L [27], RK13+E3L+K3L [27], and BSC1 cells (ATTC# CCL-26) were cultured in Dulbecco's Modified Eagle Medium (DMEM; Life Technologies) supplemented with 5% fetal bovine serum (FBS; Gemini BioProducts), and penicillin-streptomycin (Gemini BioProducts). RK13+E3L cells were grown in media supplemented with 500 μg/mL G418 (Gemini BioProducts). RK13+E3L+K3L cells were grown in media supplemented with 300 μg/mL zeocin (Life Technologies) and 500 μg/mL G418 (Gemini BioProducts). All cell lines were maintained at 37°C incubators (5% CO_2_) and split when cells reached approximately 80% confluency.

### Viruses and infections

The following viruses were used during the course of this work: wild-type ECTV (Moscow strain), ECTV (Moscow strain) expressing GFP [28], VACV (Western Reserve strain) expressing GFP (BEI resources NR-624), wild-type VACV (Copenhagen strain), VACVΔE3L (Copenhagen background), VACVΔK3L (Copenhagen background), and VACVΔE3LΔK3L (Copenhagen background). The mutant VACV strains, kindly provided by Bertram Jacobs, were constructed using homologous recombination techniques and express green fluorescent protein (GFP); more detailed information concerning these virus can be found in the previously published report by Brennan, et al [29].

With respect to wild-type VACV (Copenhagen), VACVΔE3L, VACVΔK3L, and VACVΔE3LΔK3L, infections were performed in duplicate on confluent monolayers of RK13 wild-type or RK13+E3L+K3L cells in six-well plates at a low initial multiplicity of infection (MOI = 0.01). Following one hour of incubation, the inoculum was removed, and the cells were washed twice with 1x PBS and replaced in complete media. Virus lysates were collected ∼30 hours post infection and subjected to three freeze-thaw cycles. Each virus titer was quantified using a standard serial dilution plaque assay on RK13+E3L+K3L cells and staining with crystal violet.

For single-cycle growth curves, cells were seeded into six-well plates and confluent monolayers were infected (MOI = 5) for one hour. After one hour, the infection media was removed, the cells were rinsed twice with 1x PBS, and then incubated with fresh complete medium. Samples were collected at various times post-infection and stored at −80°C. To release infectious virus, the samples were freeze-thawed three times in liquid nitrogen and a 37°C water bath followed by sonication for one minute. For virus quantification using a standard plaque assay, virus was added to monolayers of BSC1 cells by serial dilution in triplicate. After one hour of infection with each dilution, the BSC1 cells were rinsed twice with 1x PBS, and then incubated with fresh complete medium containing 1% carboxymethylcellulose (low viscosity; Sigma-Aldrich) for 2 to 5 days (2 days for VACV and 5 days for ECTV). Plaques from each dilution were observed following a crystal violet stain of the monolayers.

### Fluorescence microscopy

Fluorescent microscopy for immunofluorescence was carried out using a Zeiss Axiostar plus epifluorescent microscope and images were captured with an Optronics camera system. Cells were grown on sterile glass coverslips, infected (MOI = 5) with ECTV, and fixed at the indicated time points with 10% formalin for 10 min at room temperature. For virus factory analysis, cells were then immediately mounted on a glass slide with ProLong Gold antifade reagent with 4’-6-diamidino-2-phenylindole (DAPI; Life Technologies) and allowed to cure overnight prior to visualization. For surface B5 staining, unpermeabilized cells were incubated—after fixation—in blocking buffer [1% bovine serum albumin (BSA; Gemini BioProducts) and 2% FBS (Gemini BioProducts) in 1x PBS] for 20 min and then incubated for 40 min with anti-B5 monoclonal antibody (BEI resources NR-553) diluted in blocking buffer. After three washes with 1x PBS, Alexa Fluor 568 goat anti-Mouse IgG1 (Life Technologies) diluted in blocking buffer was applied to cells for 20 min. Finally, the coverslips were mounted as described above on a glass slide with ProLong Gold antifade reagent with DAPI. A Leica DM-IL LED inverted epifluorescent microscope was used to visualize fluorescence from VACVΔE3LΔK3L infected cells. These images were then processed using QImaging software.

### Western Blot analyses

Wild-type RK13 or RK13+E3L+K3L cells were grown overnight in 6-well plates before being mock infected or infected with various VACV strains at an MOI of 5. Total protein lysates were collected at 6 hours post-infection using 1% SDS in PBS and then sonicated. A total of 20μg of protein for each sample was separated on a 10% SDS-PAGE gel and blotted onto a 0.45μm polyvinyl difluoride (PVDF) membrane. Membranes were incubated with rabbit phosphospecific antibodies directed against Ser51 in eIF2α (BioSource International) or polyclonal rabbit anti-eIF2α (Santa Cruz Biotechnology) antibodies followed by incubation with goat anti-rabbit IgG, IgM, IgA antibodies conjugated to horseradish peroxidase (Open Biosystems). Specific binding was detected using enhanced chemiluminescence and mean band intensities were quantified with the Kodak Image Station 4000MM software. The ratios of phosphorylated eIF2α to total eIF2α for each sample were calculated from two independent western blots and the average ratios and standard deviations were determined using Microsoft Excel.

### Flow cytometry

Flow cytometry data was collected using a FACSCalibur instrument (BD Biosciences) equipped with both a red and blue laser. The collected data were analyzed using FCS Express 4 Flow Cytometry (De Novo Software; version 4.07). In experiments examining the spread of virus, 5×10^5^ cells were seeded into six-well plates. After the cells adhered to the surface, they were infected (MOI = 0.01) with GFP-expressing ECTV or VACV as described above. After 24, 48, or 72 hours, the cells were harvested using trypsin digestion. Virus spread in the culture was quantified by measuring the percentage of total cells that were positive for GFP expression. To measure surface expression of B5 protein, anti-B5 monoclonal antibody (BEI resources NR-553) was added to cells that had been harvested using trypsin treatment and washed twice with 1x PBS containing 1% BSA (Gemini BioProducts). After 30 minutes of incubation at room temperature, the cells were washed once followed by the addition of anti-mouse IgG FITC (eBioscience) for another 30 minutes. Next, to measure intracellular expression of E3 protein, cells were permeabilized using the Cytofix/Cytoperm kit (BD Biosciences) according to the manufacturer’s instructions. After permeabilization, anti-E3 monoclonal antibody (BEI resources NR-4547) was added to the cells. After 30 minutes of incubation at room temperature, the cells were washed once with Perm/Wash Buffer (BD Biosciences) followed by the addition of anti-mouse IgG2a APC (eBioscience) for an additional 30 minutes. Levels of phosphorylated eIF2α were measured in permeabilized cells (approximately 12 hours post-infection) using a monoclonal antibody (Abcam) that recognizes phosphorylated serine at amino acid residue #51; the secondary antibody used in these experiments was DyLight 488 (Abcam).

## Results

### RK13 cells do not support robust replication of ECTV

**Fig 1:**
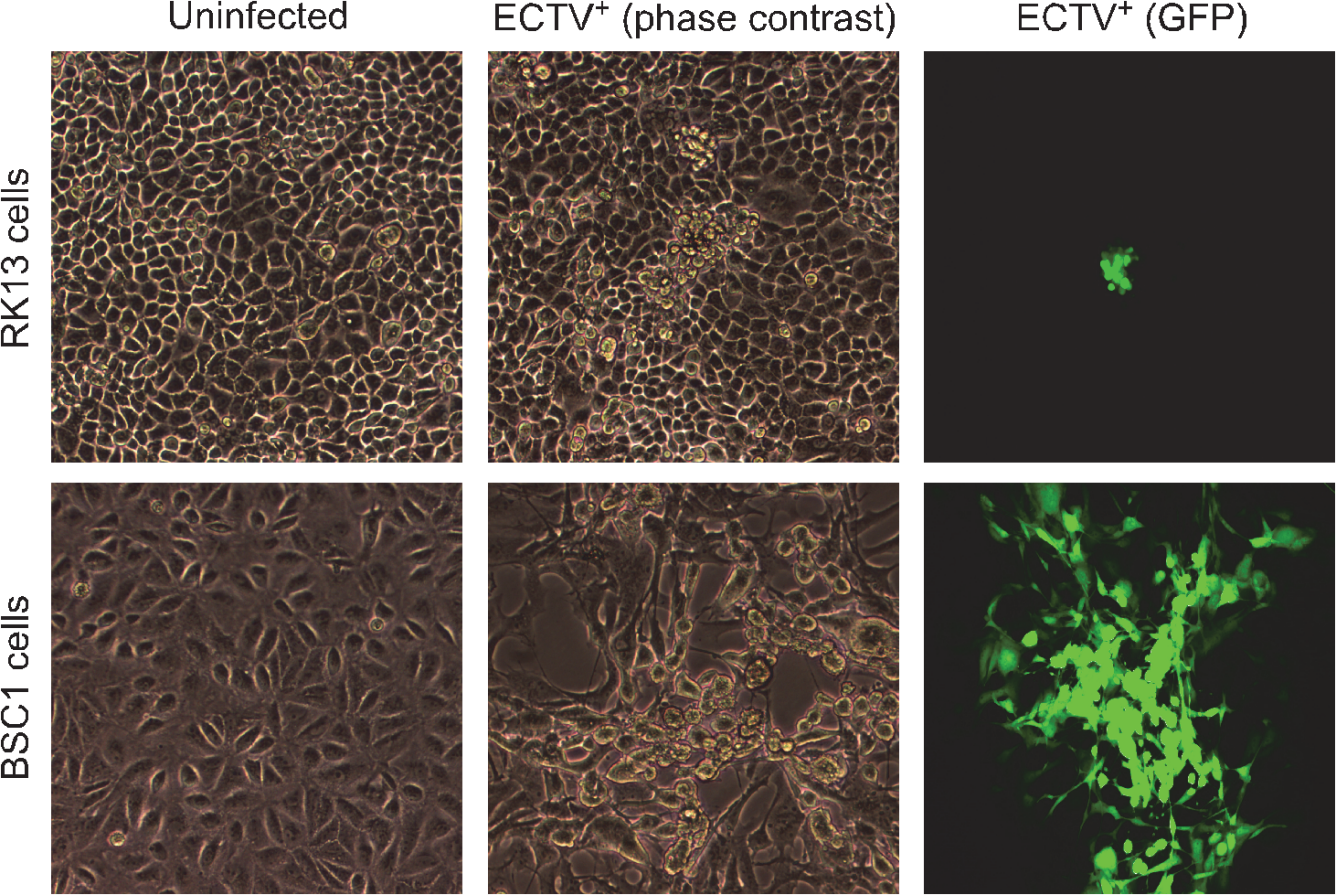
ECTV does not form plaques on RK13 cells. Monolayers of BSC1 and RK13 cells were initially infected with a low amount of ECTV-GFP (MOI = 0.001). On day 5 post-infection, the monolayers were examined for the formation of plaques. As expected, ECTV formed plaques on BSC1 cells (a representative example is shown on the bottom row). However, no plaque formation was observed on RK13 cells (a representative example is shown on the top row). All images are at 100x total magnification.

The major goal of this study was to determine the viral factors(s) that influence the ability of ECTV to replicate in cells derived from non-murine species. For example, ECTV replicates robustly in BSC1 cells (African green monkey origin) and forms clearly defined plaques on monolayers of these cells by day 5 post-infection (Fig. 1). Conversely, ECTV does not form plaques on monolayers of RK13 cells [European rabbit (*Oryctolagus cuniculus)* origin] within the same time frame (Fig. 1). For this part of the study, ECTV expressing GFP was used to track the progress of infected cells over time. We observed the formation of small GFP-positive foci, but these did not “open up” to become a plaque in the classical sense of the term—even when the infection was allowed to proceed to day 7 (data not shown). Interestingly, the closely related VACV does readily form plaques in both BSC1 and RK13 cells (data not shown). Therefore, ECTV appears to replicate inefficiently in RK13 cells and demonstrates greatly reduced ability to spread among cells in culture.

### Ectopic expression of VACV E3 and K3 rescues the replication of VACVΔE3LΔK3L in RK13 cells

As stated earlier, E3 and K3 are known to be important host range factors for various poxviruses. Since VACV can productively infect RK13 cells while ECTV cannot, we speculated that the presence of E3 and K3 from VACV in these cells may allow ECTV to replicate efficiently. Additionally, other work from us suggests that VACV K3 is a potent inhibitor of European rabbit PKR (Peng and Rothenburg unpublished observation; manuscript in preparation). Therefore, because ECTV lacks a functional K3L ortholog, we hypothesized that ECTV replication in rabbit cells might be aided by the presence of VACV K3.

**Fig 2:**
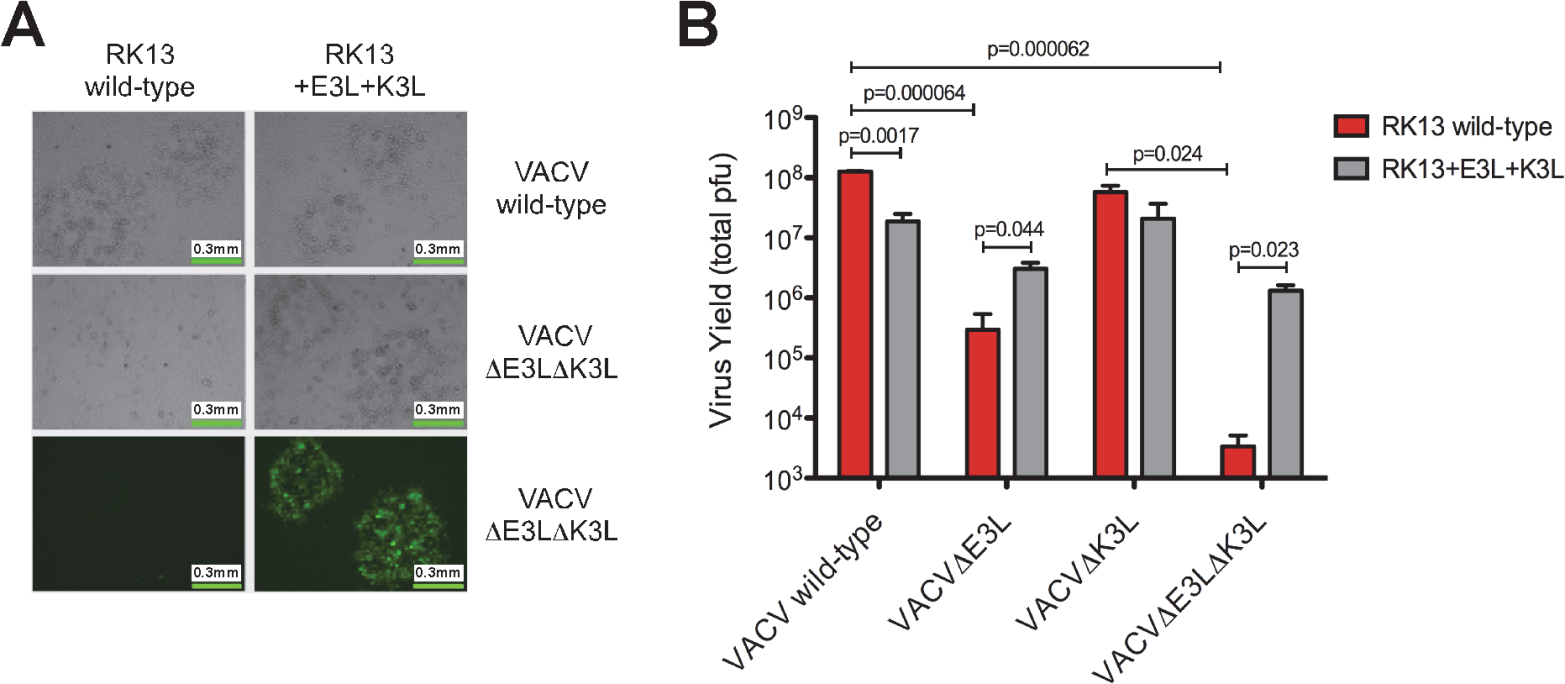
Growth of VACVΔE3LΔK3L is restored in RK13 cells expressing VACV E3 and K3 proteins. **(A)** VACVΔE3LΔK3L was constructed using homologous recombination techniques and engineered to express GFP in place of the E3L gene. No plaque formation was visualized in wild-type RK13 cells whereas replication capacity was restored in RK13 cells stably expressing E3 and K3 proteins derived from VACV. Representative images are shown. **(B)** The total yield of VACV wild-type, VACVΔE3L, VACVΔK3L, and VACVΔE3LΔK3L was determined using wild-type RK13 (red bars) and RK13+E3L+K3L (gray bars) cells. Cells were initially infected with a low amount of virus (MOI = 0.01). Virus was harvested 30 hours post-infection and the total yield was determined using standard plaque assays with RK13+E3L+K3L cells. The data is the average of two independent experiments with error bars representing the standard deviation. P-values were determined in Microsoft Excel using the Student’s t-test.

We made use of two different cell lines, RK13+E3L and RK13+E3L+K3L, that were developed and used for a previously published work [27]. These cell lines constitutively express VACV E3 and K3 proteins (data not shown). As proof of concept, we tested the ability of the RK13+E3L+K3L cells to rescue the replication of a mutant VACV (Copenhagen strain background; the virus contains GFP in the E3L locus) that has deletions in both the E3L and K3L genes [29]. The parent virus (wild type VACV-Copenhagen) produced large plaques in both RK13 wild type cells and those stably expressing E3L and K3L (Fig. 2A). However, the double-mutant (VACVΔE3LΔK3L) was only able to produce observable plaques in the RK13+E3L+K3L cells (Fig. 2A). The presence of E3L and K3L in these cells also allowed the formation of GFP-positive plaques after infection with the VACVΔE3LΔK3L virus, which indicates this virus is able to replicate in these cells. In agreement with the observed fluorescence and cytopathic effect, infection of wild-type RK13 cells with the wild-type VACV yielded a significantly higher virus titer than VACVΔE3LΔK3L, which was unable to replicate above the level of input virus (10^4^ pfu/mL; Fig. 2B). Viral replication of wild-type VACV in the RK13+E3L+K3L cells was repeatedly observed to be significantly reduced compared to replication in the wild-type RK13 cells, which may be due to a slower growth of these cells compared to the unmanipulated RK13 cells. Notably, significant replication of VACVΔE3LΔK3L occurred in RK13+E3L+K3L cells (approximately 100-fold increase over input levels).

To determine the individual contributions of E3L and K3L to VACV replication in RK13 cells, we used two additional mutant VACV strains that have deletions in either E3L or K3L alone (both viruses are Copenhagen strain background; the E3L deleted virus contains GFP in the E3L locus) and compared their replication to that of wild-type VACV and VACVΔE3LΔK3L (Fig. 2B). The VACVΔE3L virus replicated to significantly lower titers than wild-type VACV in RK13 wild-type cells, although still much higher than VACVΔE3LΔK3L (approximately 90-fold higher). VACVΔK3L, on the other hand, reached titers similar to wild-type VACV and replicated significantly better than VACVΔE3LΔK3L in these cells. In the RK13+E3L+K3L cells, both the VACVΔE3L and VACVΔK3L viruses were able to replicate similarly to the wild-type VACV, however, we consistently observed that replication of both the VACVΔE3LΔK3L and VACVΔE3L viruses in RK13+E3L+K3L cells was lower than that of wild type VACV (around 10-fold lower), although these differences were not statistically significant. It is possible that expression of E3L from the RK13+E3L+K3L cells may not be as high as during virus infection and higher expression of E3L is required to fully complement its deletion from the virus.

### Ectopic expression of VACV E3 and K3 suppresses ECTV- and VACV-induced phosphorylation of eIF2α

**Fig 3:**
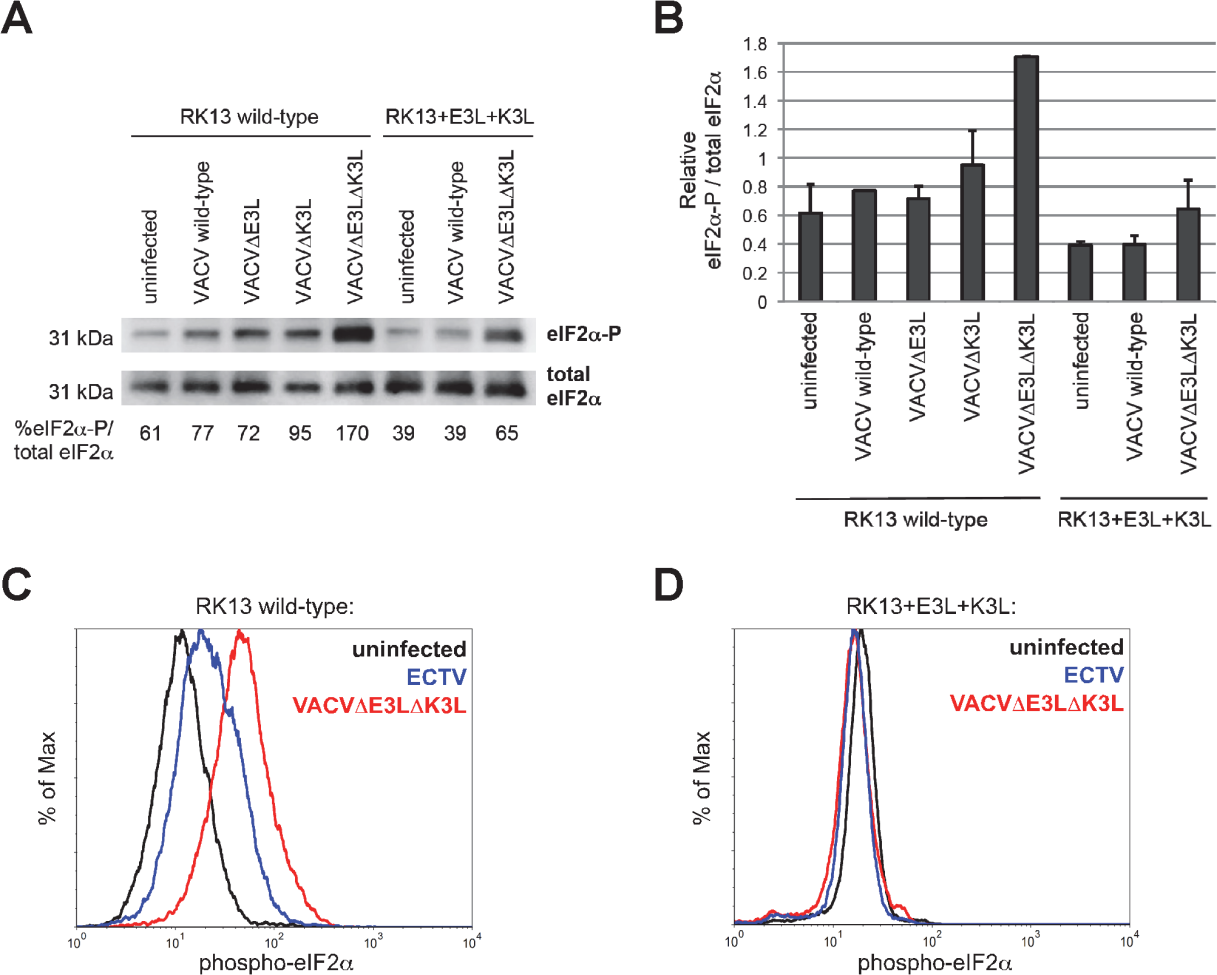
ECTV- and VACV-induced phosphorylation of eIF2α in RK13 cells. **(A)** Wild-type RK13 or RK13+E3L+K3L cells were mock infected or infected with the indicated VACV strains at an MOI of 5. Cell lysates were collected 6 hours post infection and subjected to SDS-PAGE and immunoblot analysis. Membranes were first probed with antibodies against eIF2α phosphorylated at Ser51 (eIF2α-P; top row), stripped, and then re-probed with anti-eIF2α antibodies (total eIF2α; bottom row). Band intensities were measured using the Kodak Image Station 4000MM and the ratios of phosphorylated eIF2α to total eIF2α were calculated as a percentage and listed below the panels. **(B)** The average ratios of two independent western blots are indicated in the bar graph with error bars indicating the standard deviation. **(C and D)** Wild-type RK13 **(C)** or RK13+E3L+K3L cells **(D)** were mock infected or infected with the indicated viruses at an MOI of 5 for 12 hours at which time cells were collected, permeabilized, and stained for phosphorylated eIF2α. The histograms depict relative fluorescence intensity among total cells and are representative of three independent trials.

E3L and K3L protein products are inhibitors of the cellular PKR pathway and their expression is required to inhibit the phosphorylation of eIF2α by PKR following VACV infection in RK13 cells (Fig. 3A and 3B). In wild-type RK13 cells, infection with VACVΔE3LΔK3L resulted in increased PKR activity as indicated by the higher levels of phosphorylated eIF2α whereas the wild-type VACV was able to inhibit most of this activity (Fig. 3A; top panel, compare the first and second lanes). This inhibition was further pronounced in the RK13+E3L+K3L cells for the wild-type VACV as no increase in eIF2α phosphorylation was observed relative to the uninfected cells. It is notable that the overall levels of phosphorylated eIF2α are lower in the RK13+E3L+K3L cells, which is most likely due to the activity of E3L and K3L. However, their expression in these cells was not able to completely inhibit the phosphorylation of eIF2α during infection with VACVΔE3LΔK3L (Fig. 3A; lane 7 vs. lane 8), which may explain the slight reduction in replication observed for this virus in the RK13+E3L+K3L cells compared to the wild-type. Nevertheless, the proportion of phosphorylated eIF2α relative to total eIF2α never exceeded that seen in the wild-type RK13 cells infected with wild-type VACV (Fig. 3B). These data suggest that the virus is still able to replicate successfully below a certain threshold for PKR activity, which is prevented from being reached following infection with VACVΔE3LΔK3L due to the activity of E3L and K3L in the RK13+E3L+K3L cells. Overall, we observed a negative correlation between the levels of phosphorylated eIF2α and the replicative ability of the VACV strains.

In a series of complementary experiments, we used flow cytometry to measure levels of phosphorylated eIF2α after infection with VACVΔE3LΔK3L. Consistent with the western blot results described above, this mutant virus induced robust phosphorylation of eIF2α in wild-type RK13 cells (Fig. 3C), which was inhibited in RK13+E3L+K3L cells (Fig. 3D). After infection with ECTV, there was a small but reproducible increase in phosphorylated eIF2α in wild-type RK13 (Fig. 3C) but not in RK13+E3L+K3L cells (Fig. 3D). The presence of measurable eIF2α phosphorylation after ECTV infection of wild-type RK13 cells may be related to the fact that ECTV does not have a functional K3L gene—and seemingly relies solely on E3L—while VACV does maintain a functional copy of K3L.

### Ectopic expression of VACV E3 and K3 does not enable productive ECTV infection of RK13 cells

**Fig 4:**
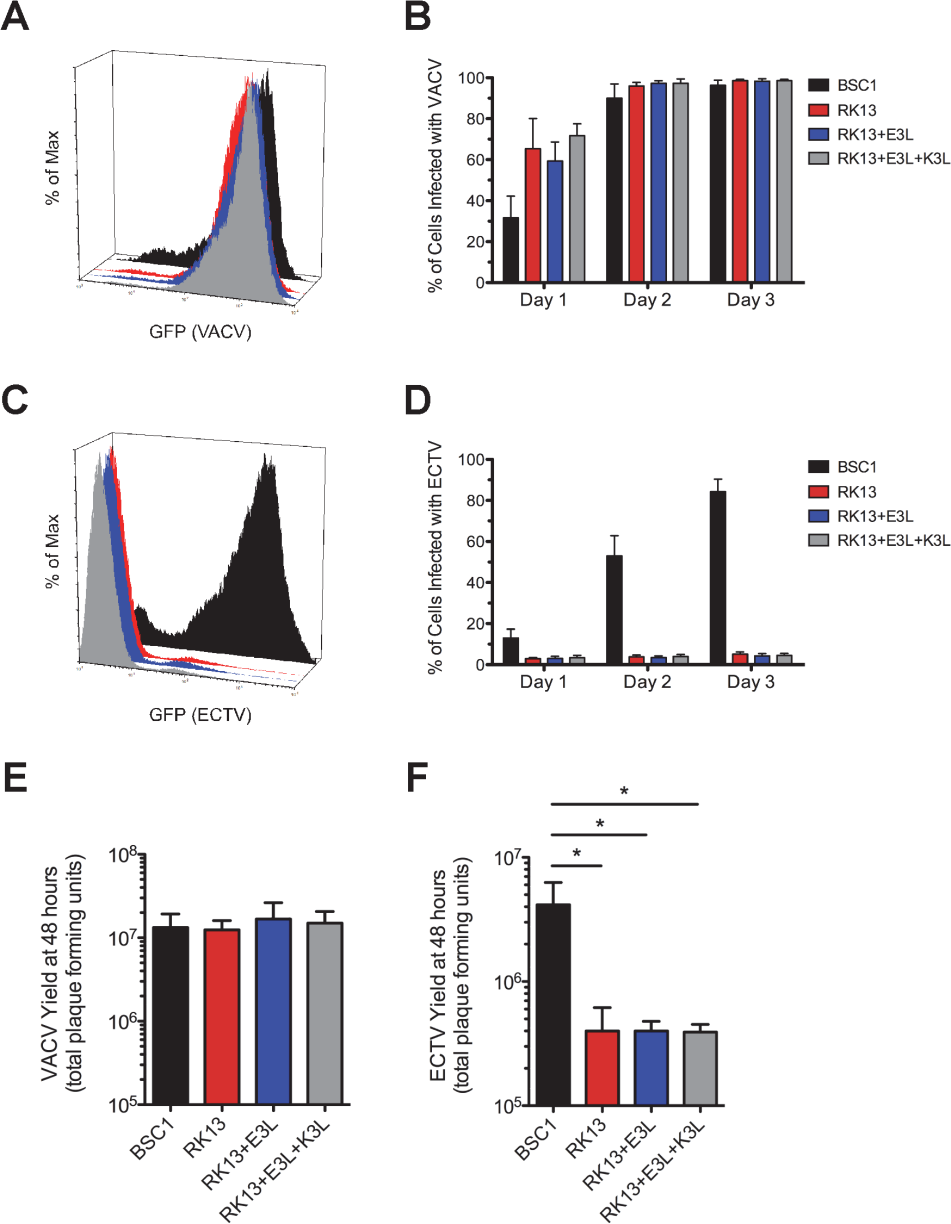
The replication of ECTV is reduced in RK13 compared to BSC1 cells even when VACV E3L and K3L are present during infection. BSC1 (black bars), RK13 (red bars), RK13-E3L (blue bars), and RK13-E3L+K3L (gray bars) cells were initially infected with a low amount of VACV-GFP (MOI = 0.01) **(A and B)** or ECTV-GFP (MOI = 0.01) **(C and D)**. Spread of virus in the culture well was quantified using flow cytometry. The histograms **(A and C)** depict relative fluorescence intensity among total cells at day 3 post-infection. The bar graphs **(B and D)** quantify levels of GFP on days 1, 2, and 3 post-infection in each cell type. The bars depict the average values and the error bars represent the standard deviations of three independent trials. The yield of w.t. VACV **(E)** and ECTV **(F)** was determined 48 hours after initial infection with a high amount of virus (MOI = 5) on cell monolayers. For quantification using a standard plaque assay, harvested virus was added to monolayers of BSC1 cells after serial dilution. The bars depict the average values and the error bars represent the standard deviations of three independent trials. Statistical analysis [performed using GraphPad Prism software (version 5.0a)] was carried out using a one-way ANOVA (nonparametric; Kruskal-Wallis) followed by a Dunns test for multiple comparisons. * denotes a p value <0.05.

In our next series of experiments, we measured the ability of VACV and ECTV to spread in cultured cells after infection with a low initial inoculum (MOI = 0.01). We used BSC1 cells as a positive control for both viruses. VACV infection spread efficiently in all tested cell lines over the course of a three day period (Fig. 4A and 4B) and ECTV displayed a similar ability to spread in BSC1 cells (Fig. 4C and 4D). Interestingly, there was no measureable difference in the replication of ECTV in any of the RK13 lines (Fig. 4C and 4D). We also conducted single-cycle growth curves as an additional means to assess virus replication. The yield of VACV was indistinguishable in all cell lines as before (Fig. 4E). ECTV demonstrated significantly greater replication in BSC1 cells compared to all RK13 lines (Fig. 4F). Moreover, there was no detectable difference in the extent of replication of ECTV in wild-type RK13 compared to the cells that constitutively express E3 by itself or with K3 (Fig. 4F).

### ECTV experiences an abortive infection in RK13 cells

**Fig 5:**
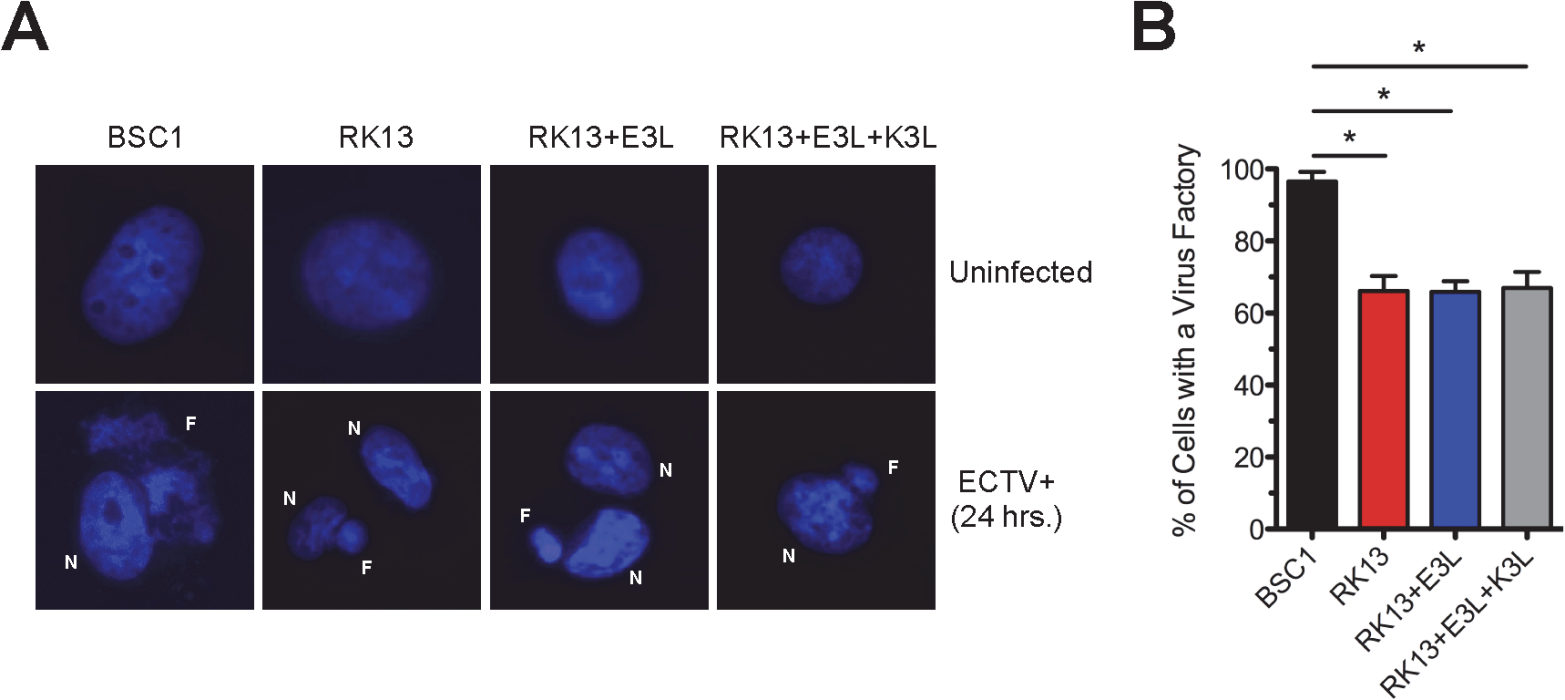
Virus factory formation during ECTV infection is more robust in BSC1 compared to RK13 cells. **(A)** Cells were grown on sterile glass coverslips, infected (MOI = 5) with ECTV, and fixed 24 hours later. Cell nuclei (N) and virus factories (F) were visualized following DAPI staining. Representative images are shown for both uninfected and infected cells. All images are at 1,000x total magnification. **(B)** One hundred randomly viewed cells within each infection condition were scored as either being positive or negative for the presence of a virus factory as compared to the uninfected control. The bars depict the average values and the error bars represent the standard deviations of three independent trials. Statistical analysis [performed using GraphPad Prism software (version 5.0a)] was carried out using a one-way ANOVA (nonparametric; Kruskal-Wallis) followed by a Dunns test for multiple comparisons. * denotes a p value <0.05.

Based on our data in this study, it appears that ECTV essentially does not complete its full replication cycle in RK13 cells. To investigate this further, we determined where in the viral replication cycle the apparent block occurs. First, we stained uninfected and infected cells with DAPI, which stains double-stranded DNA, to label host cell nuclei and to detect the presence of virus factories. Poxviruses display an entirely cytoplasmic replication cycle and, therefore, virus factories can be visualized with DAPI staining after viral DNA replication has occurred [30]. After a 24 hour infection (MOI = 5) with ECTV, virus factories were clearly discernable in BSC1 cells, which served as the positive control (Fig. 5A). In the three RK13 lines, cytoplasmic DAPI staining was observed in some cells but the relative size of the virus factories was noticeably smaller as compared to factories seen in BSC1 cells (Fig. 5A). Upon quantification (Fig. 5B), a significantly higher proportion of infected BSC1 cells (approaching 100%) contained virus factories compared to the infected RK13 lines (approximately 65% on average).

**Fig 6:**
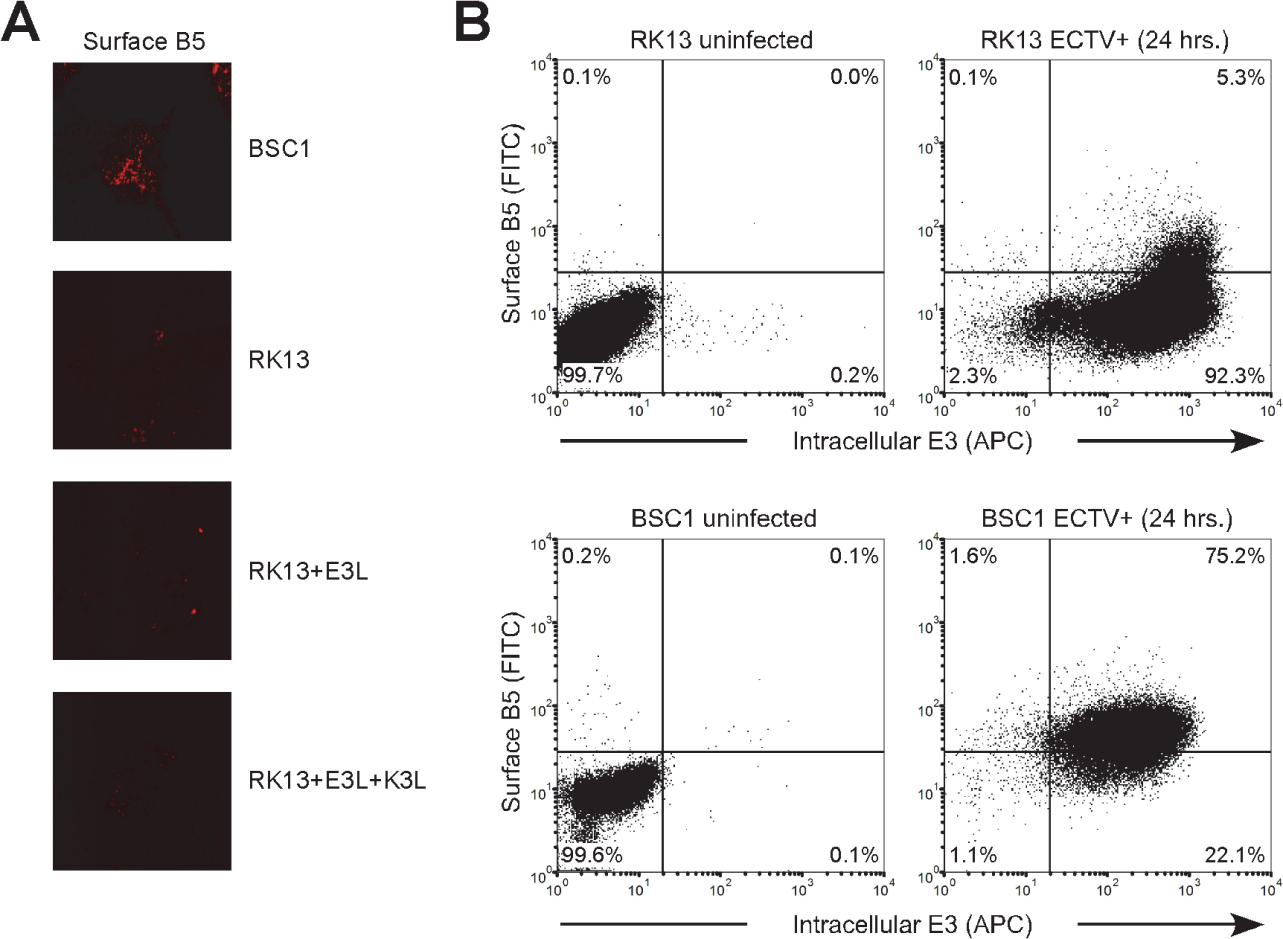
Late replication cycle events and cell surface virion assembly of ECTV are diminished in RK13 cells. **(A)** Cells were grown on sterile glass coverslips, infected (MOI = 5) with ECTV, fixed 24 hours later, and then stained for the presence of cell surface B5 protein. Representative images are shown only for infected conditions because staining of uninfected control cells yielded no detectable signal. All images are at 400x total magnification. **(B)** Cells, which had been infected (MOI = 5) for 24 hours with ECTV, were stained for surface B5 and intracellular E3 following the protocol outlined in the Methods section. All data shown are representative of three independent experiments.

Next, using fluorescent microscopy in conjunction with flow cytometry, we stained ECTV-infected cells for the presence of intracellular E3 (early gene product) and surface (non-permeabilized) expression of B5 protein, which is indicative of the presence of cell-associated/extracellular enveloped virus particles [31,32]. It should be noted that while the anti-B5 and anti-E3 antibodies were originally raised against the VACV version of these proteins, both antibodies are cross-reactive with the ECTV B5 and E3 homologues, respectively (data not shown). As shown in Fig. 6A, we could readily detect B5 on ECTV-infected BSC1 cells but very little on any of the RK13 lines. We then used flow cytometry to quantify cell surface levels of the B5 protein. A large fraction (≥75%) of BSC1 cells stained positive for B5 at 24 hours post-infection, whereas only a limited number (≤5%) of RK13 cells were positive for B5 (Fig. 6B; representative results are shown for only the wild-type RK13 cells but similar results were also obtained for the RK13+E3L and E3L+K3L lines). Thus, the block in ECTV replication in RK13 cells appears to occur sometime before the formation of mature enveloped virions at the cell surface.

Finally, we measured intracellular levels of E3 protein in ECTV-infected cells as a surrogate marker of early gene expression. As depicted in Fig. 6B, nearly 100% of all BSC1 and wild-type RK13 cells expressed ECTV-E3 protein at 24 hours post-infection (note that E3 staining is not shown for the RK13+E3L and E3L+K3L lines because both constitutively express high levels of VACV-E3 protein, even in uninfected cells). Overall, these data indicate that ECTV can enter and begin early gene expression in RK13 cells, but other downstream replication events, such as DNA replication or egress of enveloped virions, are significantly restricted or blunted (Figs. 5 and 6).

## Discussion

An interesting feature of viruses in the subfamily *Chordopoxvirinae* is that even closely-related species of the same genus can vary substantially in the range of host species that can be productively infected [11,13]. For example, ECTV and VACV are two members of the genus *Orthopoxvirus* that display disparate host range characteristics. In this work, we focused on the two previously identified VACV host range genes, E3L and K3L. The protein products of these genes are both involved in interfering with various segments of the host cell PKR pathway [16]. To give an indication of the importance of either E3L or K3L, prior work has shown that knocking out either from the VACV genome renders the virus incapable of replicating in some cell types [18].

Our data show that ECTV is unable to productively replicate in European rabbit RK13 cells in culture. Conversely, VACV can replicate efficiently in these cells. Therefore, we sought to determine if VACV E3L could rescue the replication defect of ECTV in RK13 cells. While the VACV and ECTV versions of E3 are highly related, the amino acid sequences are not completely conserved. Therefore, it is formally possible that one ortholog of E3 could be more functional in certain cell types compared to the other. Additionally, many poxviruses, including VACV, encode for a K3 ortholog. Interestingly, ECTV does not possess an intact K3L gene due to the presence of a premature stop codon and frame shift-causing deletions in the open reading frame, thus yielding a predicted truncated form of the expressed protein [12,19,25]. Since ECTV does not produce a functional K3L protein product, we hypothesized that the presence of VACV K3L in RK13 cells could then allow for the productive infection of these cells by ECTV.

Despite our initial predictions, the data presented in this study do not show a detectable role for either VACV E3L or K3L in improving the ability of ECTV to productively infect RK13 cells. In several assays, we failed to observe any significant differences with respect to the replication of ECTV in wild-type RK13 compared to versions of this cell line that stably express VACV E3 alone or with K3. Given that unpublished data has shown that K3L from VACV is a potent inhibitor of European rabbit PKR (Peng and Rothenburg; manuscript in preparation), these results were somewhat surprising. This indicates that ineffective PKR inhibition by ECTV is likely not involved in restricting its replication in RK13 cells. Meanwhile, we confirmed that E3L and K3L are required for VACV replication in RK13 cells as the VACVΔE3LΔK3L virus is unable to replicate in these cells, but can replicate efficiently in RK13 cells stably expressing these two viral genes.

The individual role of each viral inhibitor was further assessed with single deletion mutants of VACV, which showed that VACVΔE3L was slightly more attenuated than VACVΔK3L in wild-type RK13 cells. Because of this, we reasoned that while both E3L and K3L are important for VACV replication in RK13 cells, E3L may be of greater importance to virus replication in these cells. VACV expression of E3L in the absence of K3L is sufficient to allow VACV replication in wild-type RK13 cells, whereas K3L alone enables only moderate virus replication. While PKR inhibition likely does not play a role in the failure of ECTV to replicate in RK13 cells, K3L and E3L are both important for suppressing PKR phosphorylation of eIF2α during VACV infection. Indeed, our results show that the successful replication of VACVΔE3LΔK3L in the RK13+E3L+K3L cells is most likely due to the activity of the E3L and K3L gene products, which suppress PKR activation below a certain threshold that would otherwise prevent virus replication.

There is an ordered cascade of events that take place in a cell after infection with a poxvirus. First, early genes are expressed, followed by DNA replication, then late gene expression, and finally virus assembly [33]. Our data indicate that the addition of ECTV to RK13 cells largely results in an abortive infection. We provide evidence to suggest that ECTV entry is not impaired during infection of RK13 cells nor is early gene expression compromised. However, DNA replication is somewhat diminished as revealed by the significantly lower frequency of observed cytoplasmic virus factories. Furthermore, we find that the formation of enveloped virus particles at the cell surface—as measured by staining for B5 [31,32,34]—is severely blunted in RK13 cells. Therefore, a host factor (or factors) in rabbit cells is likely having a negative impact on the ability of ECTV to replicate its genome and form mature enveloped virions at the cell surface. Furthermore, it is also conceivable that the expression of viral late genes is inhibited but this possibility was not formally examined in this study.

To our knowledge, only one published paper to date [26] has shown a rescue of ECTV in RK13 cells. In this study, insertion of the Chinese hamster ovary host range gene (CHOhr/CP77) from cowpox virus into the ECTV genome extended its host range in tissue culture. This recombinant ECTV was shown to replicate more efficiently in both CHO and RK13 cells compared to wild-type virus [26]. In our study, we attempted to identify other poxvirus genes that would also endow ECTV with the capacity to replicate productively in RK13 cells. We discovered that the expression of VACV E3L and K3L in the RK13 cell line does not improve the replication capacity of ECTV. Compared to cowpox virus or VACV, ECTV has a narrow host range. In fact, the mouse is the only known host of ECTV [35]. Therefore, there are many unanswered questions related to the factor(s) that limit the host range of ECTV. Further studies are required to determine if other poxvirus gene(s) besides CHOhr/CP77 [26] can improve the ability of ECTV to replicate in cells derived from non-murine species.

The study of the viral and cellular events related to the host range of poxviruses is an important endeavor. For instance, poxviruses are important vaccine vectors [36,37] and have also shown promise as an anti-cancer therapy [38]. Additionally, there has been a recent surge in the number of human monkeypox infections in various parts of the world [39]. Therefore, the scientific and medical communities would benefit from an increased understanding of how poxviruses replicate in different cell types and the factors that limit replication in certain host species.
